# Distortion and Signal Loss in Medial Temporal Lobe

**DOI:** 10.1371/journal.pone.0008160

**Published:** 2009-12-03

**Authors:** Cheryl A. Olman, Lila Davachi, Souheil Inati

**Affiliations:** 1 Departments of Psychology and Radiology, University of Minnesota, Minneapolis, Minnesota, United States of America; 2 Department of Psychology and Center for Neural Science, New York University, New York, New York, United States of America; University of Granada, Spain

## Abstract

**Background:**

The medial temporal lobe (MTL) contains subregions that are subject to severe distortion and signal loss in functional MRI. Air/tissue and bone/tissue interfaces in the vicinity of the MTL distort the local magnetic field due to differences in magnetic susceptibility. Fast image acquisition and thin slices can reduce the amount of distortion and signal loss, but at the cost of image signal-to-noise ratio (SNR).

**Methodology/Principal Findings:**

In this paper, we quantify the severity of distortion and signal loss in MTL subregions for three different echo planar imaging (EPI) acquisitions at 3 Tesla: a conventional moderate-resolution EPI (3×3×3 mm), a conventional high-resolution EPI (1.5×1.5×2 mm), and a zoomed high-resolution EPI. We also demonstrate the advantage of reversing the phase encode direction to control the direction of distortion and to maximize efficacy of distortion compensation during data post-processing. With the high-resolution zoomed acquisition, distortion is not significant and signal loss is present only in the most anterior regions of the parahippocampal gyrus. Furthermore, we find that the severity of signal loss is variable across subjects, with some subjects showing negligible loss and others showing more dramatic loss.

**Conclusions/Significance:**

Although both distortion and signal loss are minimized in a zoomed field of view acquisition with thin slices, this improvement in accuracy comes at the cost of reduced SNR. We quantify this trade-off between distortion and SNR in order to provide a decision tree for design of high-resolution experiments investigating the function of subregions in MTL.

## Introduction

As neuroscientists and psychologists begin to ask more detailed questions regarding the functional organization of substructures in the medial temporal lobe (MTL), more sophisticated and accurate high-resolution imaging of these structures is desirable. (For reviews regarding structure and function of MTL subregions, which include the amygdala, hippocampal subfields and entorhinal, perirhinal and parahippocampal cortices, see [1], [2], [3], [4].) Unfortunately for these studies, the geometry of the brain and skull around inferior temporal regions means that image distortion and signal loss due to susceptibility-induced magnetic field gradients can be quite large and hamper interpretability of conventional resolution data. For example, the amygdala is located near the sphenoid sinus, where the difference in the magnetic susceptibilities of brain tissue and air generate unwanted gradients in the local magnetic field [5], [6]. In general, functional imaging artifacts can be minimized by fast imaging techniques (e.g. parallel imaging), but these often are available at the cost of reduced signal-to-noise ratio (SNR).

**Figure 1:**
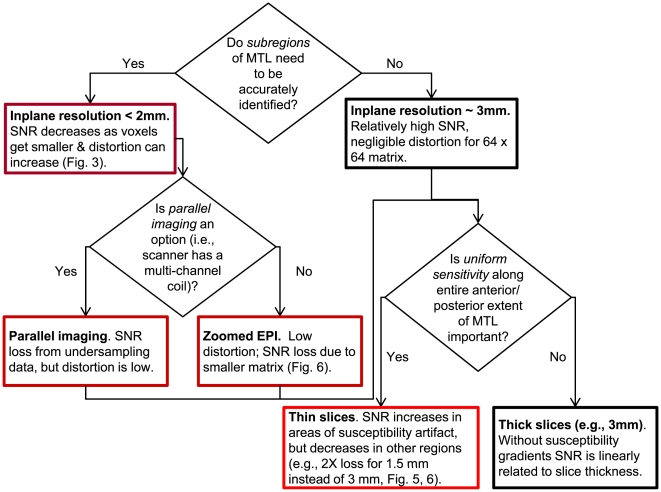
A flow chart summarizing distortion, drop-out, SNR, and coverage trade-offs for imaging in medial temporal lobe. SNR is greatest for large voxels (black box borders) and decreases when in-plane resolution is increased (dark red borders), data acquisition time is decreased (medium red borders), or slice thickness is decreased (bright red border). The optimum SNR/distortion/drop-out trade-off depends on the specific demands of the experiment.

Many excellent papers have addressed techniques for combating susceptibility artifacts in fMRI [7], [8], [9], [10], [11]; the work reported here focuses on quantifying effects in medial temporal lobe. Not all subregions of MTL are equally affected by EPI image artifacts. This means that the optimal balance between distortion and SNR will be different for each experiment. While some lateral and anterior regions of the temporal lobe are dominated by strong susceptibility-induced gradients [12], the problems in much of the MTL – including the hippocampus and large portions of the underlying parahippocampal gyrus – are not severe [13]. Here we report a systematic investigation of the severity of these susceptibility artifacts across MTL subregions, with the goal of providing a decision tree that will better inform decisions about when to sacrifice SNR in exchange for minimizing distortion or susceptibility-induced signal loss (Figure 1). The goals of this paper are: (1) to characterize which substructures of MTL are most affected, (2) to study the variability of distortion and signal-loss in MTL subregions across subjects, and finally (3) to evaluate the success of one approach in combating distortion and signal loss in MTL.

### The basic physics of distortion and signal loss

Both signal distortion and signal loss in echo planar imaging (EPI) are due to non-uniformities in the static magnetic field (B_0_). Signal distortion is due to offsets of the mean B_0_ within a voxel and increases linearly with read-out time [14], [15]. The imaging gradients in MRI encode spatial position according to resonant frequency. If the offset of the mean B_0_ in a voxel is nonzero, the MR signal from that voxel has a different frequency than expected, and this error in resonant frequency leads to a phase error that accumulates as the raw image data are recorded. As a result, image reconstruction algorithms “place” the voxel at the wrong, or distorted, image location. The greater the resonance frequency shift of the signal from a voxel and the longer it takes to read out the raw image data, the greater the error in localization of the signal (i.e., distortion is greater). Distortion can be reduced by decreasing the read-out time – either by increasing image acquisition speed or by reducing the field of view in the phase encode direction. Reducing the field of view in the phase encode direction, known as zooming, trades off geometric distortion against brain coverage [16], [17].

The distortion described above does not occur when EPI data are acquired with an alternative acquisition strategy: spiral, rather than rectilinear, EPI [8]. In this case, the effect of magnetic field distortions is image blurring rather than signal displacement. The work described herein focuses, however, on the more commonly used rectilinear EPI.

Signal loss in gradient echo EPI, commonly referred to as drop-out, is due to the variation of the static magnetic field B_0_ within a voxel. Signal drop-out increases with echo time (TE), slice thickness, and voxel size [18]. For each voxel, the variation of B_0_ within the voxel leads to a variation in the phase of each component of the MRI signal from that voxel. This phase variation results in destructive interference between the component signals and a concomitant reduction of the total received signal from that voxel [19]. Drop-out is reduced by decreasing slice thickness or increasing in-plane image resolution.

### The trade-off among distortion, drop-out, and SNR

The ability of an experiment to detect BOLD changes is determined by the contrast-to-noise ratio (CNR): the magnitude of the BOLD signal change (contrast) relative to stimulus-independent fluctuations in the BOLD signal. Noise in a BOLD experiment comes from two primary sources: physiological processes in the brain and vasculature, and thermal noise in the electronics [20], [21]. The relative contribution of physiological and thermal noise to the total noise in an fMRI experiment is resolution-dependent. At low resolution (>4 mm) the signal from most voxels is large relative to the level of noise in the electronics (i.e., thermal SNR is high) and physiological noise is dominant. However, at high resolution (<2 mm) or with thin slices, thermal SNR decreases and thermal noise can dominate [21].

Physiological noise has many sources (e.g., respiration and cardiac artifacts, motion, vasoregulatory processes and uncontrolled cognitive factors) and is strongly correlated in space and time. In general, it is difficult to reduce physiological noise, although careful design of the fMRI paradigm may be able to reduce the component of this physiological noise that is due to uncontrolled cognitive factors. Thermal noise, on the other hand, is (i) inherent in the MR coil and scanner receiver electronics,(ii) uniformly distributed throughout the image, and (iii) uncorrelated in space and time. Thermal noise can be reduced by averaging over space or time: (1) over space, by reducing the in-plane imaging resolution or increasing the slice thickness, (2) over time, by increasing the time spent acquiring each image, i.e., decreasing the bandwidth of the digital receiver, or (3) over time, by acquiring multiple images and averaging them together.

Because the relative contributions of thermal and physiological noise depend on resolution, any consideration of strategies to increase resolution or decrease artifacts must consider the impact on SNR. Fast imaging to minimize distortion requires either high receiver bandwidth or smaller data matrices, both of which decrease SNR. Similarly, the higher in-plane resolutions and thinner slices that minimize the problem of intra-voxel dephasing [5], [22] also decrease the overall SNR in the image because voxel volume (available signal) is decreased. Drop-out can also be reduced by decreasing the echo time (TE), but decreasing the echo time (TE) decreases sensitivity to the BOLD effect [23] and thus negatively affects the CNR.

We describe here a systematic study of the separate contributions of signal distortion and drop-out to the quality of high-resolution EPI images in the MTL. We find that while distortion and drop-out seriously degrade image quality in lateral regions of inferior temporal cortex, these artifacts are less of a problem in MTL. The hippocampus itself suffers from no distortion. Only the anterior parahippocampal gyrus, including portions of the entorhinal and perirhinal cortex, suffers significantly from susceptibility artifacts. A high-resolution, zoomed field-of-view acquisition greatly reduces susceptibility artifacts in parahippocampal gyrus, but at the cost of reduced SNR.

## Methods

Experiments were carried out on a 3 Tesla Allegra scanner (Siemens, Erlangen, Germany) equipped with a volume transmit/receive birdcage head coil (Nova Medical, Wakefield, MA, USA).

### Subjects

6 subjects (3 female, age 25 to 35) participated in the experiments. The experimental protocols conformed to safety guidelines for MRI research and were approved by the Institutional Review Board at New York University.

### Reference anatomy

To define an undistorted anatomical reference, a 3D MP-RAGE volume covering the entire head was acquired with 1 mm isotropic resolution.

### Functional MRI

All EPI image volumes were acquired in an oblique coronal orientation, perpendicular to the hippocampal axis. A 2D, navigated, single-shot, zoomed field of view EPI pulse sequence with interleaved slice ordering was used for all fMRI scans. This sequence is a 2D version of that reported in [17]. Outer volume suppression was turned on only for the zoomed acquisition (see below).

For the conventional low-resolution EPI (3×3×3 mm voxels) the field of view (FOV) was 19.2×19.2×8.7 cm; matrix size = 64×64×29; TR = 2000 ms; TE = 25 ms; echo-spacing = 0.32 ms; total read-out time: 20.5 ms. Conventional, high-resolution EPI (1.5×1.5×2 mm voxels): FOV = 19.2×19.2×5.8 cm; matrix size = 128×128×29; TR = 2000 ms; TE = 37 ms (minimum achievable with the Allegra head-only gradient system); echo-spacing = 0.52 ms; total read-out time: 66.4 ms. Zoomed, high-resolution EPI (1.5×1.5×2 mm voxels): FOV = 19.2×4.8×5.8 cm; matrix size = 128×32×29; TR = 2000 ms; TE = 25 ms; echo-spacing = 0.52 ms; total read-out time: 16.6 ms. For the zoomed, high-resolution data acquisition, the field of view was reduced in the phase encode direction (Foot-Head) and the signal from tissue superior and inferior to the volume of interest was suppressed in a manner similar to [16], [17]. Each TR therefore consisted of a slab-specific saturation pulse for outer volume suppression (OVS), a frequency-selective fat saturation pulse, a slice selective excitation pulse, three navigator echoes, a ramp-sampled rectilinear EPI trajectory, and spoiler gradients after the readout. The volume transmit coil used in these experiments provided a spatially uniform B_1_ field and allowed for efficient OVS. For all scans, the raw data were reconstructed off-line using custom C and Matlab code. For each slice B_0_ and pre-phase ghost corrections were computed based on the navigator data [24], [25]. Within scan motion correction was not performed because subjects were experienced and scans were only 1 minute each, resulting in negligible head motion.

For each type of EPI image, 30 timepoints were acquired while the subject was resting (no task). To estimate image signal-to-noise ratio in each ROI, each voxel's mean intensity was divided by its standard deviation through time. Only the last 25 timepoints were used, discarding the first 5 timepoints to avoid data during which signal intensity was changing because magnetization was not yet at steady-state.

### Alignment to volume anatomy

For each data set (conventional low-resolution, conventional high-resolution and zoomed high-resolution), EPI data were aligned directly to the 3D MP-RAGE volume. Automatic image alignment was accomplished using a robust intensity-based algorithm [26]. Before aligning the EPI volumes directly to the 3D MP-RAGE volume, a mean image was formed by averaging the first 30 timepoints together. The contrast of the mean EPI image was inverted to match the contrast of the T_1_-weighted reference anatomy: a tissue mask was formed by thresholding the mean EPI image intensity (above and below), then subtracting the intensity of each voxel from the sum of the image maximum and the image minimum. Much more sophisticated histogram inversion algorithms are available; alternatively, alignment based on mutual information removes the need for contrast inversion [27], but this was sufficient for successful, automatic alignment of the EPI data to the volume anatomy.

### Field mapping and voxel displacement maps

To measure the field in inferior temporal lobe, a multi-echo FLASH sequence was used to acquire 32 echoes for each read-out line. The sequence was identical to the zoomed EPI sequence, except a separate excitation was used for each phase encode line, and phase-encoding blips were absent during the read-out train [28]. TR = 2000 ms; TE = 5.20, 6.22 … 21.84 ms; total acquisition time = 64 s. For the first two data sets, the Fieldmap toolbox distributed with SPM2 (http://www.fil.ion.ucl.ac.uk/spm/) was used to calculate a frequency offset map, using as input two pairs of phase and magnitude images acquired at 5.20 and 7.28 ms (high-resolution). For the last four data sets, field maps were calculated with custom in-house software; a comparison of the two methods indicated little difference. From the frequency offset maps, corresponding voxel displacement maps were calculated for appropriate acquisition parameters: at each voxel location, the frequency offset was divided by the pixel bandwidth in the phase encode direction (1/T_RO_, where T_RO_ is the total read-out time).

### Region of interest definition

Seven bilateral regions of interest were defined separately in each subject: anterior hippocampus, middle hippocampus, posterior hippocampus, entorhinal cortex, perirhinal cortex, posterior parahippocampal gyrus, and the amygdala. Anatomical ROIs were defined by the criteria given by Pruessner *et al*. [29]. Briefly, parahippocampal gyrus was defined as the cortex extending from just medial to the hippocampus through the lateral bank of the collateral sulcus. It was divided into three subdivisions. At a point roughly 4 mm posterior to the gyrus intralimbicus and continuing in an anterior direction until the appearance of the fronto-temporal junction, the lateral bank of the collateral sulcus was marked as perirhinal cortex. The area medial to the collateral sulcus was marked entorhinal cortex, and the posterior boundary was the gyrus intralimbicus (4 mm anterior to the posterior boundary of PRC) [29]. The parahippocampal cortex posterior to the perirhinal border and ending at the same posterior border as the hippocampus was labeled as posterior parahippocampal cortex. The anterior boundary of the hippocampus, at the border with the amygdala, was marked by the appearance of the uncal recess of the inferior horn of the lateral ventricle [30]. The hippocampus itself was divided along the A/P direction into three parts: anterior, middle and posterior, based on subjective judgment of the apparent width of the hippocampus. The amygdala was defined as the medial gray matter extending from a posterior limit defined by the uncal recess of the inferior horn of the lateral ventricle to an anterior limit defined the frontotemporal junction.

## Results

**Figure 2:**
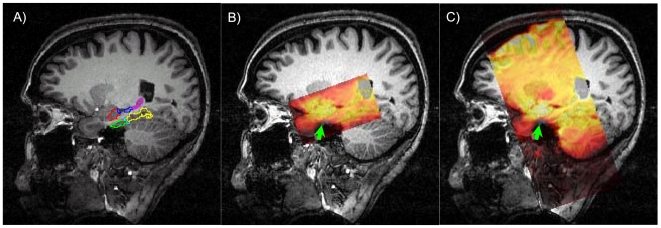
Region of interest definition and alignment of zoomed and full field of view (FOV) EPI with reference 3D MP-RAGE volume anatomy. (A) ROI definitions, shown on a parasagittal section: red = anterior hippocampus (aHIPP); blue = middle hippocampus (mHIPP); magenta = posterior hippocampus (pHIPP); green = entorhinal cortex (ER); cyan = perirhinal cortex (PR: not seen in this section); yellow = posterior parahippocampal gyrus (PHG). (B) Location of zoomed EPI volume (after automated alignment); EPI images are shown as a partially transparent overlay with a hot colormap. Signal loss due to through-slice gradients is apparent where gray matter in anterior parahippocampal regions is visible on the underlying anatomical image (green arrow). (C) For full FOV, high-resolution EPI volume increased distortion is evident in anterior parahippocampal gray matter (slice thickness and in-plane resolution are equated, so drop-out is equivalent for EPI data shown in (B,C). Low-resolution EPI data (not shown) covered the same field of view as the high-resolution EPI data shown in (C).

To quantify effects of distortion on subregions in MTL, regions of interest were delineated on the 3D MP-RAGE volume data set for each subject and then translated to the EPI data and field map acquired during the same scanning session. Each EPI and field map scan was aligned directly to the reference 3D volume anatomy using a robust intensity-based alignment procedure ([26]; results for one subject are shown in Figure 2). Directly aligning each data set to the reference anatomy eliminated any potential errors due to head motion. Distortion was then assessed by voxel displacement maps calculated from the field map data; signal loss was quantified by the measured SNR in each of the EPI acquisitions.

**Figure 3:**
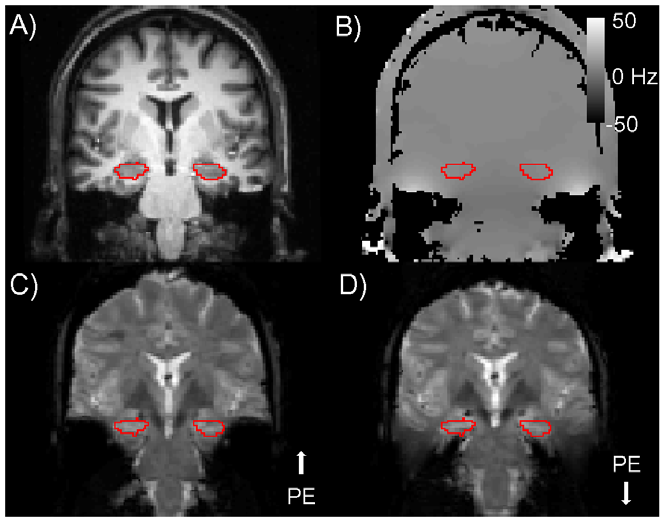
Reversing the direction of the phase-encode gradients reverses the direction of distortion. (A) Reference anatomical image for an oblique coronal slice (perpendicular to hippocampal axis) through anterior hippocampus (red boundary). (B) Field map (colorbar indicating frequency offsets in Hz) for same the slice; ROI boundary is the same as in (A). (C) High resolution, full field of view EPI image with phase-encode (PE) in the Foot-Head direction. Susceptibility artifacts decrease the static magnetic field in medial temporal lobe and shift signal from PHG toward the feet (notably, hippocampus is not affected). Increased B_0_ (static magnetic field strength) in lateral temporal lobe shifts signal toward the top of the head. (D) Reversing the phase-encode direction reverses the direction of distortion, pushing PHG signal up into hippocampus. The choice of phase encode direction can affect localization accuracy when fieldmap-based distortion compensation techniques are used during data analysis.

An important note concerns the effect of phase-encode direction on image distortion, demonstrated qualitatively in Figure 3. Field distortions are small in medial temporal lobe, but large in lateral temporal lobe (Figure 3B). For a positive field distortion, signal is shifted in the direction of the phase-encode gradient blips, which can be arbitrarily selected as Foot-Head or Head-Foot. With a Foot-to-Head phase encode direction, signal from medial temporal lobe was displaced in an inferior direction while signal in lateral temporal cortex was mapped onto more superior cortex (Figure 3C); reversing the phase-encode direction reverses the direction of the distortion and stretches the signal out (Figure 3D). With sufficient SNR, these opposite effects can have different implications for subsequent data analysis, as discussed at the end of this paper. It is important to note also that the hippocampus itself (red outline) is relatively unaffected by field inhomogeneities.

**Figure 4:**
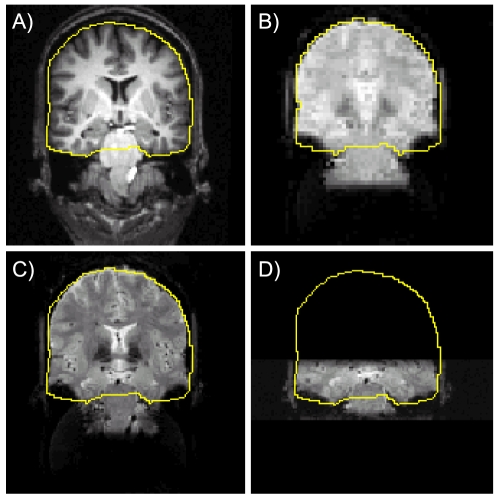
Signal drop-out is worse in low-resolution images because thicker slices result in more signal loss due to drop-out, and distortion is worse in full FOV high resolution images due to long read-out times. (A) Reference anatomy; yellow line is for visual reference and the same in all 4 panels. (B) Conventional low resolution EPI (3 mm isotropic voxels; total read-out, T_RO_ = 22.5 ms; echo time, TE = 25 ms). Signal from lateral inferior temporal lobe is missing. (C) Full FOV high resolution image (T_RO_ = 66.6 ms; TE = 37 ms). A combination of signal displacement and signal loss affects lateral temporal cortex. Note tissue signal from medial temporal cortex extends bilaterally below fiducial lines. (D) Zoomed FOV image (T_RO_ = 16.6 ms; TE = 25 ms). Both distortion and signal loss are minimized.

**Figure 5:**
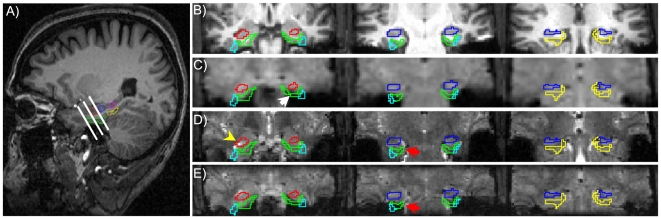
Distortion and signal loss due to through-slice dephasing are evident in the most anterior regions of parahippocampal gyrus (PHG), while the posterior half of the hippocampus (HIPP) and PHG are unaffected by susceptibility artifacts. (A) Location of slices and ROIs for a representative subject, shown on parasagittal section of the reference anatomy (3D MP-RAGE). (B) Three slices resampled from reference anatomy in anterior MTL to match location and resolution of functional data (zoomed EPI). Red and blue regions of interest are anterior and middle hippocampus; cyan (PR), green (ER) and yellow (pPHG) regions of interest are parahippocampal gyrus. (C) Low resolution EPI acquisition. Thicker slices (3 mm) result in increased signal loss due to through-slice dephasing in PHG (white arrow). (D) High-resolution full FOV EPI images. In the most anterior slice, field gradients in the hippocampus displace signal so the ventricle, rather than the hippocampus, is in the selected region of interest (yellow arrow). Similarly, PHG ROIs contain signal from HIPP (red arrow), and PHG signal is lost. (E) Zoomed high-resolution EPI images. Signal is lost only in anterior PHG (ER and PR); distortion is negligible.

Predictably, distortion was greatest in the full field of view (full FOV) high-resolution acquisition, because these images required the longest read-out time (Figure 4C). On the other hand, signal loss due to intra-voxel dephasing was most severe in the low-resolution data because of the thicker slices and larger voxels (Figure 4B). In all three EPI acquisitions, however, only the data from anterior regions of MTL were affected by distortion and signal loss (Figure 5). Importantly, the zoomed images acquired using a 2 mm slice thickness and outer volume suppression (OVS) to minimize the image acquisition time showed the least effect of either distortion or signal loss due to intra-voxel dephasing in anterior PHG.

**Figure 6:**
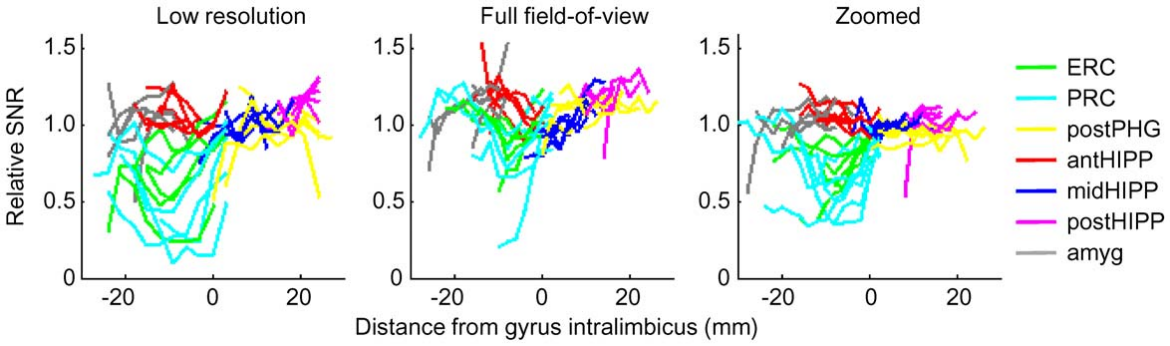
Individual variation of drop-out: zoomed (6 subjects), full FOV (5 subjects), and low resolution EPI (5 subjects). Average signal-to-noise ratio (SNR) in each slice of each ROI, normalized by the average SNR in posterior hippocampus (where no signal loss is present). Slice position is measured from gyrus intralimbicus (G.I.), with negative distances indicating anterior regions. SNR is calculated as mean intensity divided by the standard deviation of 25 timepoints. In anterior PHG, SNR could be either unaffected by intra-voxel dephasing or decreased by as much as 75%, depending on the individual.

Figure 6 illustrates the loss of signal due to intra-voxel dephasing in anterior regions of MTL, as well as the consistency of the data in regions posterior to the gyrus intralimbicus. To compare SNR along the length of the hippocampal axis, average SNR was calculated in each slice of each ROI in each subject, then normalized by the average SNR in the posterior hippocampus ROI (the ROI least affected by susceptibility artifacts). Data for individual subjects are shown for each acquisition technique in Figure 6. There is notable variability between subjects, as might be expected from differences in anatomy both on a large scale (position of cerebellum and brainstem relative to inferior temporal cortex and length of hippocampal axis) and on a small scale (thickness of PHG and size of hippocampus). We found that signal loss was most severe in low-resolution acquisitions because of the increased slice thickness, so these data sets showed the least consistency in SNR along the hippocampal axis. SNR appears more consistent for full FOV than for the zoomed high-resolution acquisition but this is because distortion can mask signal drop-out by shifting signal from the HIPP into the PHG ROI (red arrow, Figure 5D). This observation – the ease with which signal from more superior regions may be misinterpreted as originating from PHG – underscores the importance of short read-out times for studies in which localization is important. This error is also observed in Figure 5D (yellow arrow) where signal from the inferior horn of the lateral ventricle is displaced into a hippocampal ROI.

**Figure 7:**
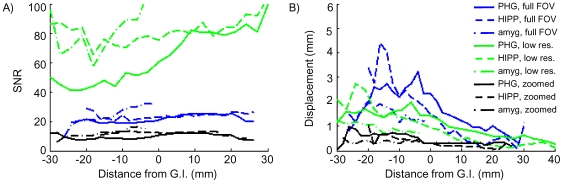
Comparison of SNR and distortion: zoomed, full FOV, and low resolution EPI. (A) SNR, averaged across subjects, as a function of position along the hippocampal axis. A centimeter posterior to the gyrus intralimbicus (GI), SNR follows the expected relationships. First, comparing the two high-resolution acquisitions: the reduced FOV (zoomed) acquisition has the same resolution as the full FOV high-resolution acquisition, but only a quarter of the data points. Because the Fourier transform takes a weighted average of all raw data points to calculate the intensity of each image pixel, the thermal SNR in the final image is directly related to the square root of the number of points in the raw data matrix (assuming equivalent, uncorrelated thermal noise in the source data). Therefore an SNR reduction by a factor of 2 is expected (and observed) for the reduced FOV acquisition (solid lines), relative to the full FOV (dot-dash lines) acquisition. Second, comparing low- and high-resolution data: the low-resolution acquisition has a voxel volume 6 times greater than the high-resolution full FOV acquisition, increasing available signal by 6X, but only 1/4 the data points are acquired for each image (64×64 matrix instead of 128×128), for a √4 reduction in thermal SNR and a net 3X increase in SNR (low-resolution > high-resolution). (B) Average voxel displacement for each of the 3 EPI acquisitions, calculated from the fieldmap for each slice of each ROI. All three acquisitions were acquired in the same scanning session for each subject, so the field distortions are identical in each case. Voxel shift is linearly related to total read-out time, which is shortest for the low-resolution data. But voxels are larger in the low-resolution acquisition, so total displacement is smallest in the zoomed, high-resolution acquisitions.

Quantifying both distortion and signal-to-noise ratio on a slice-by-slice basis for each EPI acquisition illustrates the trade-off between local SNR and distortion: an acquisition that minimizes distortion has low SNR. In Figure 7A, average SNR for all subjects (rather than the normalized SNR for individual subjects of Figure 6) is plotted for the HIPP and PHG ROIs for the three methods tested. (To estimate the average loss across all subjects, data were aligned at the gyrus intralimbicus, an anatomical landmark in the hippocampus that was used to set the posterior boundaries of entorhinal cortex and perirhinal cortex during manual ROI definition.) In the relatively artifact-free regions of posterior HIPP,PHG and amygdala, the expected SNR relationships are seen: low-resolution > full FOV > zoomed. However, even though the high-resolution acquisitions have lower inherent SNR than the low-resolution acquisition, the SNR (data quality) is more consistent throughout the length of the hippocampal axis. Average SNR and noise values, for all ROIs in all subjects, are given in Table 1.

**Table 1:** Average noise (standard deviation of signal intensity in voxels in regions of interest), signal (mean signal intensity), and signal-to-noise ratio for each acquisition technique.

	zoomed	full FOV	low-resolution
noise	120 (6)	258 (18)	191 (20)
signal	1414 (310)	5662 (1147)	12883 (3737)
SNR	12 (3)	22 (5)	69 (22)

Values are given as mean (standard deviation, across ROIs and subjects).

The above observations were verified with a two-way analysis of variance (ANOVA) for the SNR, with acquisition technique and ROI as factors. Both factors were significant (acquisition technique: F_2,84_ = 262, *p*<0.001, ROI: F_6,84_ = 4.93, *p*<0.001), as was the interaction (F_12,84_ = 2.32, *p* = 0.013). The interaction was further investigated with separate one-way ANOVAs for the three acquisition techniques, testing variability between ROIs. SNR variation between ROIs was significant for the low-resolution data (F_6,28_ = 3.33, *p* = 0.013), but not for the zoomed (F_6,35_ = 2.00, *p* = 0.092) or full FOV (F_6,28_ = 1.32, *p* = 0.28) data. To test whether SNR differences were due to variation in the mean signal intensity rather than local noise characteristics (noise was characterized as the standard deviation of the signal intensity), a two-way ANOVA verified a significant effect of modality on the noise (F_2,84_ = 703, *p*<0.001), but no effect of ROI (F_6,84_ = 3.33, *p* = 0.07) with an insignificant interaction (F_2,84_ = 1.2, *p* = 0.28). The main effect of noise, and therefore basic SNR in tissue without susceptibility artifact, is the consequence of different bandwidth and image matrix size for the different acquisition techniques – see Figure 7 legend for details.

In addition to providing more consistent (albeit lower) SNR than the low-resolution acquisition, the zoomed acquisition also provides more consistent localization accuracy than either the low-resolution or the full FOV high-resolution acquisitions (Figure 7B). In posterior regions of MTL (posterior PHG and middle and posterior hippocampus) we observed little distortion. Typical signal displacement in anterior MTL was less than 1 mm with the zoomed EPI acquisition, compared to 2 or 3 millimeters in the low-resolution data, and 3 or 4 millimeters in the full FOV acquisition.

**Figure 8:**
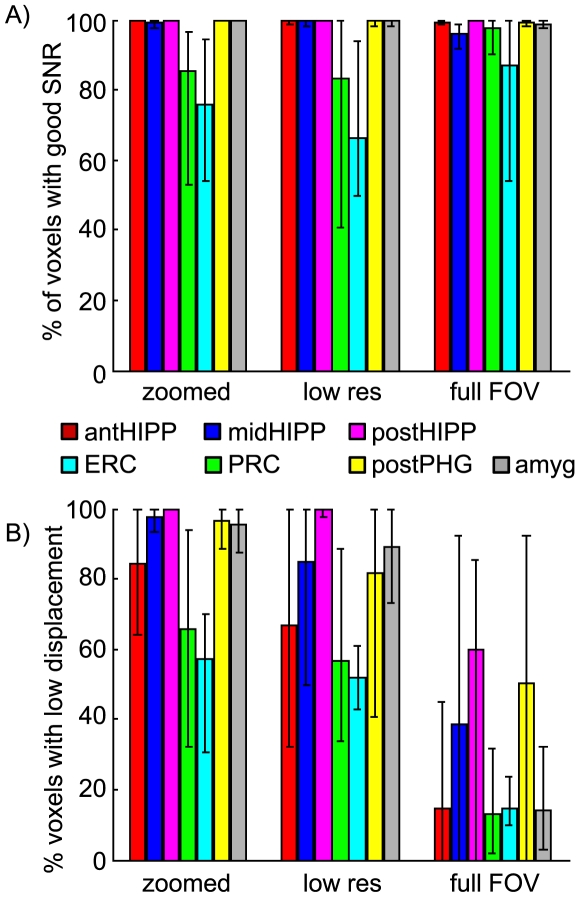
Summary of distortion and drop-out in MTL sub-regions. (A) Percent of voxels in each ROI, with each acquisition technique, in which signal was reduced by more than 50%, relative to the SNR in the middle hippocampus ROI. Proportion with signal >50% reference signal was calculated for each subject, then averaged. Errorbars indicate the range of values across subjects. (B) Percent of voxels in each ROI/acquisition in which distortion was greater than half of an in-plane voxel dimension (displacement greater than 0.75 mm for zoomed and full FOV acquisitions, 1.5 mm for low-resolution acquisition). Averages and errorbars calculated as in (A).

Summarizing the separate effects of distortion and drop-out, we quantified the percentage of each ROI in each acquisition that suffered either significant distortion (displacement by more than half a voxel) or significant drop-out (loss of more than 50% of the mean signal intensity). Results, averaged across subjects and plotted by ROI and acquisition technique, are shown in Figure 8. Black errorbars indicate the minimum and maximum values across the group of subjects. The high variability of data quality in entorhinal and perirhinal cortex is again apparent, as is the severity of distortion in the full FOV acquisition (Figure 8B).

## Discussion

The most significant findings of this study were that image quality varied substantially along the anterior-posterior extent of the parahippocampal gyrus at 3T, and the ease with which signal from superior regions of the MTL (i.e., hippocampus) were displaced into more inferior PHG regions (both illustrated in Figure 5). First, we found that while distortion and drop-out were negligible in posterior hippocampus and posterior parahippocampal gyrus regardless of the image resolution or field of view, the imaging accuracy and SNR in anterior MTL regions, particularly in the anterior parahippocampal gyrus, were strongly dependent on the particular imaging resolution and pulse sequence parameters. Second, using a conventional moderate-resolution EPI acquisition (3 mm isotropic voxels) led to a non-uniform reduction in SNR across the length of the PHG (on average, by a factor of 2 in anterior PHG relative to posterior PHG). This underscores the importance of not making direct region to region comparisons in overall BOLD activation, but instead relying on interaction effects across two experimental conditions in two different regions [31]. Finally, we found that the overall SNR was more consistent along the entire length of the PHG in high resolution acquisitions, even though it was decreased compared to conventional resolution. Importantly, this consistency in SNR (albeit lower overall) would allow researchers to avoid the possibility that an experiment would be sensitive to BOLD effects in posterior but not anterior regions of the parahippocampal gyrus.

We observed several instances in which distortion masked signal loss in anterior regions of medial temporal lobe, providing an important caveat for studies requiring accurate spatial localization in anterior MTL. With pseudo-coronal images with a foot-head phase encode direction, signal from anterior hippocampus was displaced downward. At the same time, immediately inferior to the hippocampus, strong field gradients caused signal drop-out in anterior parahippocampal gyrus. The result was that, instead of an apparent black hole in the images, the signal displaced from the hippocampus gave the impression of signal originating in the parahippocampal gyrus. If no effort were made to minimize or compensate distortion, regions of interest based on undistorted anatomical scans would be labeled “anterior PHG” but would include functional contrast originating in anterior hippocampus. This caveat applies to high-resolution studies with relatively long image acquisition times; in this study, the short image acquisition times for low-resolution data resulted in distortions always less than a voxel.

We acquired fieldmaps but did not actually apply distortion compensation to the data because we wanted to quantify the severity of distortion and signal loss in the data acquisition, without dependence on the efficacy of a particular distortion correction algorithm. Distortion compensation algorithms are readily available and distributed with most software packages, and this study reemphasizes the importance of distortion compensation for high-resolution EPI acquisitions. However, in our experience, imperfect alignment between fieldmaps and functional data, and the difficulty of accurately interpolating fieldmap data when distortion is severe, limit the utility of distortion compensation techniques for severe artifacts on the edge of the brain. This is one reason why techniques such as outer volume suppression that avoid distortion in strongly affected brain regions may be worth using, in spite of decreased signal-to-noise ratio.

The pulse sequences we used to quantify the effects of susceptibility artifacts on signal-to-noise ratio differed in several ways that would affect SNR even in the absence of distortion or drop-out. As seen in Figure 7, the basic SNR of the low-resolution acquisition is much larger than for the high-resolution acquisitions because of the voxel size. Similarly, the different matrix sizes used in the acquisition have an impact on SNR because different numbers of samples are acquired in each image acquisition (see Figure 7 legend for details). Finally, the echo time was longer in the full FOV acquisition than in the zoomed acquisition – the longer echo time (37 ms, compared to 30 ms) would have a negative impact on the SNR for the full FOV acquisition (an 8% decrease). This longer echo time was used to accommodate the large matrix size. Therefore, there are two comparisons to be made between each acquisition type. First, SNR should be compared in well-shimmed brain regions (e.g., post PHG) to judge baseline data quality. Then, consistency of SNR across brain regions should be considered (as in the normalized data of Figure 6) to predict the consistency of BOLD sensitivity across multiple regions of interest.

In discussing the effects of signal loss in MTL, we must be careful to note that functional contrast-to-noise ratio (CNR) and image signal-to-noise ratio (SNR) are not entirely equivalent concepts. It is the CNR, not the SNR, which is important for detecting BOLD signal changes. CNR is always low: to pick rough numbers, if the BOLD contrast in a typical voxel is 2%, then an SNR of better than 100 is required to achieve a CNR of 2∶1 and detect a BOLD effect in a single voxel on a single trial. As seen in Figure 7, such high SNR is rare, particularly in the middle of the brain, although for more superficial brain regions the SNR is often higher. Almost all fMRI experiments must therefore rely on averaging to overcome the intrinsically low CNR to permit reliable detection of BOLD contrast. The difference in SNR between anterior PHG and posterior PHG discussed above would imply that, roughly speaking, 30 repetitions of a trial may be sufficient for reliable measurement of BOLD contrast in posterior PHG, whereas BOLD effects in anterior PHG would go undetected with the same number of repetitions.

A natural extension of the work here is to take advantage of parallel imaging on multi-channel systems[32], [33]. With multiple receive coils, the read-out time for a high-resolution image can be reduced without sacrificing field of view (although not without sacrificing SNR). Distortion can therefore be nearly eliminated [34], leaving only the problem of intra-voxel dephasing (drop-out).

BOLD contrast is maximized by an echo time matched to the T_2_* of the tissue – approximately 40 ms [35] for gray matter at a field strength of 3 Tesla – which creates an inherent limitation in our ability to address intra-voxel dephasing. Z-shimming or the acquisition and averaging of thin slices can effectively reduce signal drop-out [22], [36], [37], [38]. These options can be costly in terms of temporal resolution, but where parallel imaging allows an increase in the number of slices acquired per second, it facilitates these approaches to compensating for intra-voxel dephasing. Asymmetric spin-echo and spin-echo pulse sequences are also effective at combating intra-voxel dephasing [39]. Spin-echo EPI has been used successfully to compensate for signal drop-out in orbitofrontal cortex [40]. Where high spatial resolution is desirable, spin-echo acquisitions are particularly attractive because they reduce the strength of the BOLD contrast from spatially inaccurate large veins, increasing the relative importance of BOLD contrast from more spatially specific small veins and capillaries [41]. At 3T, however, this component of the BOLD signal is weak [42], resulting in a low contrast-to-noise ratio for BOLD experiments.

In addition to choosing a pulse sequence protocol, an investigator has the choice of orientation for the phase-encode direction [11], [43]. In this study, the use of outer volume suppression to restrict the field of view mandated that the phase-encode direction be along the inferior/superior axis. However, we still had the choice of sampling the phase- encode direction in one of two ways: 1) head to foot or 2) foot to head. In this study, the magnetic field distortion in the MTL was always negative, while in the lateral temporal lobe the field distortion was always positive (Figure 3). Therefore, a foot to head phase encode direction resulted in displacement of the MTL signal in an inferior direction. Several of the major analysis packages provide distortion compensation as a standard post-processing analysis step: a field map acquired during the experiment is combined with knowledge of the phase-encode direction and total read-out time to unwarp the EPI images [44]. It is critical to note that preference should be made for the signal to be stretched out (like in our MTL regions), as opposed to compressed (as was the case for lateral temporal regions). When signal is stretched out, the signal from each voxel remains distinct, maximizing the accuracy of spatial localization after distortion correction. In regions of the brain where the image is compressed (as in lateral temporal lobe in Figure 5), the signal from two adjacent voxels is combined into one and, hence, cannot be separated using post-processing analyses. The best that a post-processing field map correction algorithm can do in this case is to spread the compressed signal between the two voxels. Therefore, selection of the phase-encode orientation can dictate localization accuracy for regions where susceptibility-induced artifacts are large. This specific choice of phase encoding direction for a given study will depend on what brain regions are the foci of current hypotheses.

Our data also demonstrate significant variability between subjects in the severity of distortion and drop-out in medial temporal lobe, which suggests that researchers interested in these regions should select an analysis strategy that can accommodate these significant individual differences. One such strategy is similar to our approach – define anatomically guided regions of interest in individual subjects and average data extracted from ROIs, rather than warping brains into a common space and then averaging data. Consideration of the data quality on a case-by-case basis would let an investigator eliminate subjects from the analysis based on a pre-determined SNR or distortion criterion, to avoid negatively impacting sensitivity to BOLD changes or localization accuracy.

In conclusion, high-resolution imaging of the MTL is accurate as long as the read-out time is short and slices are thin. This can be accomplished without sacrificing temporal resolution by employing either a reduced field-of-view or using parallel imaging. We found that anterior portions of the PHG are the most strongly affected by susceptibility-induced gradients in the MTL. Sensitivity to signal changes in entorhinal cortex, in particular, is poor in standard gradient echo EPI pulse sequences. However, the cost of fast, high-resolution imaging is a loss of SNR, which can compromise BOLD contrast-to-noise ratio – a limitation that requires increased experiment durations to allow for averaging additional stimulus presentations.

## References

[1] Squire L, Stark CE, Clark RE (2004). The medial temporal lobe.. Annual Reviews of Neuroscience.

[2] Davachi L (2006). Item, context and relational episodic encoding in humans.. Current Opinion in Neurobiology.

[3] Eichenbaum H, Yonelinas AP, Ranganath C (2007). The medial temporal lobe and recognition memory.. Annual Reviews of Neuroscience.

[4] Mayes A, Montaldi D, Migo E (2007). Associative memory and the medial temporal lobes.. Trends in Cognitive Sciences.

[5] Chen N-K, Dickey CC, Yoo S-S, Guttmann CRG, Panych LP (2003). Selection of voxel size and slice orientation for fMRI in the presence of susceptibility field gradients: application to imagign of the amygdala.. NeuroImage.

[6] Robinson S, Windischberger C, Rauscher A, Moser E (2004). Optimized 3 T EPI of the amygdalae.. NeuroImage.

[7] Stenger VA, Boada FE, Noll DC (2000). Three-dimensional tailored RF pulses for the reduction of susceptibility artifacts in T2*-weighted functional MRI.. Magnetic Resonance Imaging.

[8] Glover GH, Law CS (2001). Spiral-in/out BOLD fMRI for increased SNR and reduced susceptibility artifacts.. Mag Res Med.

[9] Hutton C, Bork A, Josephs O, Diechmann R, Ashburner J (2002). Image distortion correction in fMRI: a quantitative evaluation.. NeuroImage.

[10] Diechmann R, Gottfried JA, Hutton C, Turner R (2003). Optimized EPI for fMRI studies of the orbitofrontal cortex.. NeuroImage.

[11] Weiskopf N, Hutton C, Josephs O, Deichmann R (2006). Optimal EPI parameters for reduction of susceptibility-induced BOLD sensitivity losses: a whole-brain analysis at 3 T and 1.5 T.. NeuroImage.

[12] Ojemann JG, Akbudak E, Snyder AZ, McKinstry RC, Raichle ME (1997). Anatomic localization and quantitatve analysis of gradient refocused echo-planar fMRI susceptibility artifacts.. NeuroImage.

[13] Eldridge LL, Engel SA, Zeineh MM, Bookheimer SY, Knowlton BJ (2005). A dissociation of encoding and retrieval processes in the human hippocampus.. Journal of Neuroscience.

[14] Jezzard P, Balaban RS (1995). Correction for geometric distortion in echo planar images from B0 field variations.. Magnetic Resonance in Medicine.

[15] Sekihara K, Kohno H (1987). Image restoration from nonuniform static field influence in modified echo-planar imaging.. Medical Physics.

[16] Pfeuffer J, Van de Moortele PF, Yacoub E, Shmuel A, Adriany G (2002). Zoomed functional imaging in the human brain at 7 Tesla with simultaneous high spatial and high temporal resolution.. NeuroImage.

[17] Fleysher L, Fleysher R, Heeger DJ, Inati S (2005). High resolution fMRI using a 3D mutli-shot EPI sequence.. Proceedings of the International Society of Magnetic Resonance in Medicine.

[18] Farzaneh F, Riederer SJ, Pelc NJ (1990). Analysis of T2 limitations and off-resonance effects on spatial resolution and artifacts in echo-planar imaging.. Magnetic Resonance in Medicine.

[19] Weiskopf N, Hutton C, Josephs O, Turner R, Diechmann R (2007). Optimized EPI for fMRI studies of hte orbitofrontal cortex: compensation of susceptibility-induced gradients in the readout direction.. Magnetic Resonance and Material Physics.

[20] Kruger G, Glover GH (2001). Physiological noise in oxygenation-sensitive magnetic resonance imaging.. Mag Res Med.

[21] Triantafyllou C, Hoge RD, Krueger G, Wiggins CJ, Potthast A (2005). Comparison of physiological noise at 1.5 T, 3 T and 7 T and optimization of fMRI acquisition parameters.. NeuroImage.

[22] Merboldt KD, Finsterbusch J, Frahm J (2000). Reducing inhomogeneity artifacts in functional MRI of human brain activation-thin sections vs gradient compensation.. J Magn Reson.

[23] Ogawa S, Menon RS, Tank DW, Kim S-G, Merkle H (1993). Functional brain mapping by blood oxygenation level-dependent contrast magnetic resonance imaging. A comparison of signal characteristics with a biophysical model.. Biophysical Journal.

[24] Van de Moortele PF, Pfeuffer J, Glover GH, Ugurbil K, Hu X (2002). Respiration-induced B0 fluctuations and their spatial distribution in the human brain at 7 Tesla.. Magnetic Resonance in Medicine.

[25] Thesen S, Kruger G, Muller E (2003). Absolute correction of B0 fluctuations in echo-planar imaging.. Proceedings of the International Society of Magnetic Resonance in Medicine.

[26] Nestares O, Heeger DJ (2000). Robust multiresolution alignment of MRI brain volumes.. Magnetic Resonance in Medicine.

[27] Jenkinson M, Bannister PR, Brady JM, Smith SM (2002). Improved optimisation for the robust and accurate linear registration and motion correction of brain images.. NeuroImage.

[28] Fleysher R, Fleysher L, Inati S (2005). Fast direct image reconstruction for MRI and fMRI in the presence of field inhomogeneities and T2*.. Proceedings of the International Society of Magnetic Resonance in Medicine.

[29] Pruessner JC, Kohler S, Crane J, Pruessner M, Lord C (2002). Volumetry of temporopolar, perirhinal, entorhinal and parahippocampal cortex from high-resolution MR images: considering the variability of hte collateral sulcus.. Cerebral Cortex.

[30] Pruessner JC, Li LM, Serles W, Pruessner M, Collins DL (2000). Volumetry of hippocampus and amygdala with high-resolution MRI and three-dimensioinal analysis software: minimizing the discrepancies between laboratories.. Cerebral Cortex.

[31] Henson R (2005). What can functional neuroimaging tell the experimental psychologist?. Q J Exp Psychol A.

[32] Pruessmann KP (2004). Parallel imaging at high field strength: synergies and joint potential.. Topics in Magnetic Resonance Imaging.

[33] Bellgowan PSF, Bandettini PA, van Gelderen P, Martin A, Bodurka J (2006). Improved BOLD detection in the medial temporal region using parallel imaging and voxel volume reduction.. NeuroImage.

[34] Schmidt CF, Degonda N, Luechinger R, Henke K, Boesiger P (2005). Sensitivity-encoded (SENSE) echo planar fMRI at 3 T in the medial temporal lobe.. NeuroImage.

[35] Wansapura JP, Holland SK, Dunn RA, Ball WSJ (1999). NMR relaxation times in the human brain at 3.0 tesla.. Journal of Magnetic Resonance Imaging.

[36] Constable RT (1995). Functional MR imaging using gradient-echo echo-planar imaging in the presence of large static field inhomogeneities.. J Magn Reson Imaging.

[37] Cordes D, Turski PA, Sorenson JA (2000). Compensation of susceptibility-induced signal loss in echo-planar imaging for functional applications.. Magn Reson Imaging.

[38] Heberlein K, Hu X (2004). Simultaneous acquisition of gradient-echo and asymmetric spin-echo for single-shot z-shim: Z-SAGA.. Mag Res Med.

[39] Zheng J, Ehrhardt JC, Cizadlo T, Yuh WT (1997). Comparison of inversion recovery asymmetrical spin-echo EPI and gradient-echo EPI for brain motor activation study.. Journal of Magnetic Resonance Imaging.

[40] Norris DG, Zysset S, Mildner T, Wiggins CJ (2002). An investigation of the value of spin-echo-based fMRI using a Stroop color-word matching task and EPI at 3 T.. NeuroImage.

[41] Yacoub E, Van de Moortele PF, Shmuel A, Ugurbil K (2005). Signal and noise characteristics of Hahn SE and GE BOLD fMRI at 7 T in humans.. NeuroImage.

[42] Parkes LM, Schwarzbach JV, Bouts AA, Deckers RHR, Pullens P (2005). Quantifying the spatial resolution of the gradient echo and spin echo BOLD response at 3 Tesla.. Magnetic Resonance in Medicine.

[43] De Panfilis C, Schwartzbauer C (2005). Positive or negative blips? The effect of phase encoding scheme on susceptibility-induced signal losses in EPI.. NeuroImage.

[44] Jezzard P, Balaban RS (1995). Correction for geometric distortion in echo planar images from B0 field variations.. Mag Res Med.

